# Expression of tumor-associated macrophages and PD-L1 in patients with hepatocellular carcinoma and construction of a prognostic model

**DOI:** 10.1007/s00432-023-04949-y

**Published:** 2023-06-12

**Authors:** Panpan kong, Huan Yang, Qing Tong, Xiaogang Dong, Mamumaimaitijiang-Abula Yi, Dong Yan

**Affiliations:** The First Ward of Hepatobiliary and Pancreatic Surgery, Tumor Hospital Affiliated to Xinjiang Medical University, Urumqi, Xinjiang 830001 People’s Republic of China

**Keywords:** Hepatocellular carcinoma, Programmed cell death ligand 1, M1 macrophages, M2 macrophages, Immune cells, Prognosis

## Abstract

**Background:**

Hepatocellular carcinoma (HCC) is an inflammation-associated tumor involved in immune tolerance and evasion in the immune microenvironment. Immunotherapy can enhance the immune response of the body, break immune tolerance, and then recognize and kill tumor cells. The polarization homeostasis of M1 and M2 macrophages in tumor microenvironment (TME) is involved in the occurrence and development of tumors and has been considered a hot topic in tumor research. Programmed cell death ligand 1 (PD-L1) plays an important role in the polarity of TAM and affects the prognosis of HCC patients as a target of immunotherapy. To this end, efforts were hereby made to further explore the application value of PD-L1, M1 macrophages (CD86), and M2 macrophages (CD206) in the prognosis assessment of HCC, their correlation with immune cell infiltration in HCC tissues, and their bioenrichment function.

**Methods:**

The gene expression omnibus (GEO) and the Cancer Genome Atlas (TCGA) database were used to analyze the expression of PD-L1, CD86, and CD206 in different tumor tissues. The correlation between the expression of PD-L1, CD86, and CD206 and the infiltration of immune cells was analyzed using the Tumor Immune Estimation Resource (TIMER). The tissue specimens and clinicopathological data of hepatocellular carcinoma patients having undergone surgical treatment in our hospital were collected. Immunohistochemistry was used to verify the expression of PD-L1, CD86, and CD206, and analyze the relationship with clinicopathological features and prognosis of patients. Besides, nomogram was constructed to predict the overall survival (OS) of patients at 3 and 5 years. Finally, the protein–protein interaction network information was analyzed using STRING database, and GO analysis and KEGG (Kyoto Encyclopedia of Genes and Genomes) analysis were performed to study the biological functions of PD-L1, CD86, and CD206.

**Result:**

Bioinformatics analysis found that PD-L1, CD86, and CD206 were underexpressed in various tumor tissues including liver cancer, while the present immunohistochemical detection found that PD-L1, CD86, and CD206 were overexpressed in liver cancer tissues. Expressions of PD-L1, CD86, and CD206 were positively correlated with the infiltration level of immune cells in liver cancer, while the expression of PD-L1 was positively correlated with the degree of tumor differentiation. Meanwhile, the expression level of CD206 was positively correlated with gender and preoperative hepatitis, and patients with high expression of PD-L1 or low expression of CD86 had poor prognosis. AJCC stage, preoperative hepatitis, and the expression levels of PD-L1 and CD86 in cancer tissues were independent risk factors affecting survival of patients after radical hepatoma surgery. KEGG pathway enrichment analysis showed that PD-L1 was significantly enriched in T cell aggregation and lymphocyte aggregation, and might be involved in the formation of T cell antigen receptor CD3 complex and cell membrane. Besides, CD86 was significantly enriched in positive regulation of cell adhesion, regulation of mononuclear cell proliferation, regulation of leukocyte proliferation, and transduction of T cell receptor signaling pathway, while CD206 was significantly enriched in type 2 immune response, cellular response to LPS, cellular response to LPS, and involvement in cellular response to LPS.

**Conclusion:**

In conclusion, these results suggest that PD-L1, CD86, and CD206 may be involved not only in the occurrence and development of HCC, but also in immune regulation, indicating the potential role of PD-L1 and CD86 as potential biomarkers and new therapeutic targets for prognosis assessment of liver cancer.

## Introduction

Hepatocellular carcinoma (HCC) is the seventh most common cancer worldwide and the second most lethal malignant tumor after lung cancer, heterogeneous in etiology and biology, which is also the most common histological subtype (Kim and Viatour 2020; Bray et al. 2018). Tumor microenvironment (TME) is composed of tumor cells, a variety of immune cells and stromal cells, and is a special tissue structure dependent on the occurrence and development of solid tumors (Mcgiynn et al. 2021; Nasrollahzadeh et al. 2020). Tumor-associated macrophages (TAM) play an important role in the composition of TME, accounting for more than 50% of some TME cells. In terms of the function, TAM is usually divided into two types, namely, M1 type of classical activation pathway and M2 type of alternating activation pathway. The phenotype of M1 macrophages is CD86, while that of M2 macrophages is CD206, producing two opposite effects of anti-tumor or promoting tumor development through mutual transformation (Kim and Bae 2016; Zeng et al. 2019; Pan et al. 2020; Rhee 2016). It has been reported in the literature that M2 TAM is closely related to the poor prognosis of liver cancer (Arvanitakis et al. 2022). In recent years, the polarization homeostasis of M1 and M2 macrophages in TME has become a hot topic in tumor research, endowed with great significance for tumor prognosis. Meanwhile, PD-L1, a 40 kDa transmembrane protein encoded by CD274 gene, is induced to be expressed in T cells, B cells, dendritic cells, macrophages and mesenchymal stem cells, and its expression is rapidly upregulated in tumor tissues in response to interferon and other inflammatory factors (Taube et al. 2012; Keir et al. 2020; Bardhan et al. 2016). Studies have found that tumor-associated macrophages can also express programmed cell death ligand 1 (PD-L1), which matters considerably in regulating TAM polarity. Genevieve et al. found that the downregulation of PD-L1 could regulate TAM polarization and activate M1 macrophages to inhibit tumor progression in melanoma (Hartley et al. 2018). Herein, the correlation between the expression of tumor-associated macrophages CD86, CD206, and PD-L1 in hepatocellular carcinoma and clinicopathology was analyzed, and their clinical application value in the prognosis assessment of patients was highlighted.

## Materials and methods

### Expression and immunocorrelation analysis of pan-cancer in TIMER database

The TIMER (Tumor Immune Estimation Resource) database is a data analysis platform based on the TCGA (https://cistrome.shinyapps.io/timer/). The expressions of CD86, CD206, and PD-L1 in different cancers were analyzed by Diff Exp module of TIMER. Besides, correlation modules were used to calculate the correlation between the expression level of PD-L1 and CD86 and CD206 in HCC tissues, and the setting conditions included: (1) cancer type: LIHC (liver hepatocellular carcinoma); (2) gene symbols (Y-axis):PD-L1(CD274); (3) gene symbols (X-axis): CD86; (4) gene symbols (X-axis): CD206(MRC1); and (5) correlation adjusted by: tumor purity.

### Analysis of the expression of CD86, CD206, and PD-L1 in HCC tissues and adjacent tissues using TCGA database

Database of Gene Expression Profiling Inter-active Analysis (GEPIA∥gepia.Cancer-pku.cn) is a web analysis tool based on TCGA and GTEx data, which can provide differential expression analysis, contour mapping, patient survival analysis, related gene analysis, and other functions and was hereby used to analyze the expression differences between liver cancer tissues and normal tissues: (1) gene: CD86, CD206 and PD-L1; and (2) datasets selection: LIHC, keep the default values for other filters.

### Patient data and specimens

From January 2014 to December 2015, 60 patients with hepatocellular carcinoma who were treated for the first time in the Department of Hepatobiliary and Pancreatic Surgery, Affiliated Cancer Hospital of Xinjiang Medical University were hereby selected as the research objects. Their cancer tissues and paired adjacent tissues were collected during the operation. All 60 patients with liver cancer strictly met the exclusion criteria and inclusion criteria. The inclusion criteria included: (1) first radical resection of liver tumor; (2) hepatocellular carcinoma confirmed by pathology; (3) complete clinical data; and (4) no previous treatment for liver cancer; while the exclusion criteria included: (1) patients undergoing palliative surgery or unable to complete resection; (2) metastatic hepatocellular carcinoma or intrahepatic cholangiocarcinoma confirmed by pathology; (3) incomplete clinical data; (4) previously received human mediated or targeted or immune therapy. Information was collected in detail, including the patient's gender, age, history of hepatitis B, liver cirrhosis, AFP, Carcino-embryonic antigen (CEA), carbohydrate antigen 19–9, CA19-9), total bilirubin (TBIL), alanine aminotransferase (ALT), aspartate transaminase, AST albumin level, prothrombin time (PT), intraoperative blood loss, tumor maximum diameter, tumor differentiation, microvascular invasion (MVI), etc. This study was approved by the Ethics Committee of the Affiliated Cancer Hospital of Xinjiang Medical University.

### Immunohistochemical staining

The conventional paraffin samples were precooled, then sliced into 4 μm sections, dewaxed three times with xylene, hydrated in ethanol gradient, and antigen repaired with methylcitric acid (PH = 6) at high temperature and pressure for 3 min. After being cooled to room temperature, the samples were blocked for 10 min to eliminate endogenous peroxidase activity. The primary antibodies were PCNP protein (1:200) and β-catenin (1:200), respectively, which were incubated at 4℃ overnight for about 16 h. After being rewarmed for 1 h, the secondary antibodies were flushed and incubated at 26℃ for 30 min. Upon the completion of each of the above steps, the samples were washed with phosphate buffer solution (PBS) for three times. Finally, DAB was performed for color development, restaining, differentiation, gradient ethanol dehydration, xylene transparency, and neutral gum sealing. The results were observed under light microscope, and image analysis was performed. Positive control sections provided by the ordering reagent company were used for positive control, while PBS instead of the primary antibody was used for negative control.

### Result interpretation

According to the staining intensity and staining positive rate of cytoplasm and nucleus, the cancerous tissues and the adjacent tissues were interpreted, respectively. PCNP is a quantitative assessment mainly expressed in the nucleus. The presence of brown particles in the nucleus is considered a positive result, and a little of the cytoplasm is brown. β-Catenin is a qualitative assessment, which shows membrane staining in normal cells and cytoplasmic or nuclear staining in abnormal cells. Herein, five fields in each tissue section were randomly selected under high power microscope (× 200) to observe the staining degree of positive cells and calculate the percentage of positive cells. These fields were scored according to the degree of staining: 0 for non-staining, 1 for light yellow, 2 for brown, and 3 for tan. They were also scored according to the percentage of positive cells: 0 for a percentage less than 1%; 1 for 1 ~ 25%; 2 for 26 ~ 50%; 3 for 51 ~ 75%; and 4 for more than 75%. The product of "staining intensity score" and "proportion of positive cells score" was used as the total score for grouping, with a value ≤ 4 divided into the negative group and > 4 into the positive group. Two senior pathologists evaluated the final results in a double-blind manner.

### Statistical methods

The data were input into SPSS 22.0 software for statistical processing, and measurement data conforming to normal distribution were expressed as x ± s. Meanwhile, *T *test was used for comparison between two groups, while one-way analysis of variance and corresponding multiple comparisons were used for comparison among multiple groups. The count data were analyzed by Chi-square test and expressed as n (%). Besides, Kaplan–Meier method was used to draw the survival curve, and Cox proportional hazards regression model was used for univariate and multivariate analysis of survival. All statistical results were calculated, with a value of *P* < 0.05 considered statistically significant. Using the selected independent risk factors as variables, a nomogram model was established to predict the 1 -, 3 -, and 5-year specific survival rates of patients with hepatocellular carcinoma, and internal validation was performed correspondingly. The discrimination and calibration ability of nomogram was evaluated by the C-index and calibration curve. Net reclassification index (NRI) and decision curve analysis (DCA) were used to evaluate the predictive ability and net benefits of nomogram. To narrow the bias, the above analyses were repeated 1000 times with Bootstrap. A total risk score was calculated for each patient according to the prediction model, and patients in the modeling group were stratified according to the quartile of the total risk score. Meanwhile, the significance of survival differences among risk groups was evaluated using the Kaplan–Meier method and Log rank test.

## Results

### Expression of PD-L1, CD86, and CD206 in different tumors

**Fig. 1:**
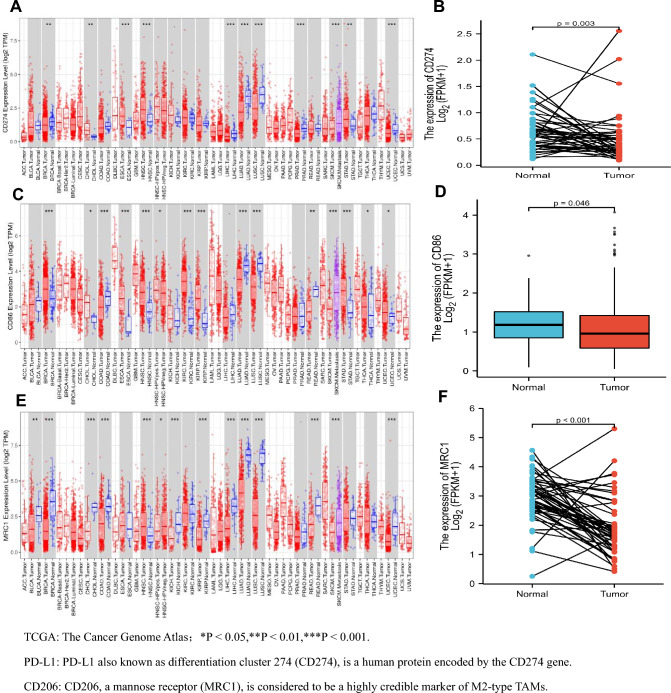
Expression levels of PD-L1, CD86, and CD206 in tumors.** A** Based on TCGA data analysis, the expression level of PD-L1 (CD274) in 38 tumor tissues (TIMER). **B** PD-L1 (CD274) expression level in liver cancer tissues and adjacent tissues (UALCAN). **C** CD86 expression level in 38 tumor tissues based on TCGA data analysis (TIMER). **D** CD86 expression level in liver cancer tissues and adjacent tissues (UALCAN). **E** Expression level of CD206 (MRC1) in 38 tumor tissues based on TCGA data analysis (TIMER). **F** CD206(MRC1) expression level in liver cancer tissues and adjacent tissues (UALCAN)

PD-L1, CD86, and CD206 have been reported to be differentially expressed in a variety of tumors. Similar results were hereby obtained through TIMER database analysis. PD-L1, also known as surface antigenin 274 (CD274), is a human protein encoded by the CD274 gene, and CD206, a mannose receptor (MRC1), is considered a highly reliable marker of M2-type macrophages. Herein, the transcriptome levels of PD-L1 (CD274), CD86 and CD206 (MRCI) were found to be lower in many tumor tissues than in adjacent normal tissues, such as bladder urothelial carcinoma (BLCA), breast cancer (BRCA), colon cancer (COAD), hepatocellular carcinoma (LIHC), lung adenocarcinoma (LUAD), lung squamous cell carcinoma (LUSC), and prostate cancer (PRAD). However, for head and neck squamous cell carcinoma (HNSC), the transcriptome levels of PD-L1, CD86, and CD206 in tumor tissues were higher than those in adjacent normal tissues, as shown in Fig. 1A, C, and E. Expression analysis in liver cancer tissues showed that the expression levels of PD-L1, CD86, and CD206 in liver tissues were significantly lower than those in paracancer tissues, presenting statistical significance (*P* = 0.003, *P* = 0.046, *P* < 0.001), as shown in Fig. 1B, D, and F.

### Relationship between PD-L1, CD86, and CD206 expression and immune cell infiltration

**Fig. 2 Relationship:**
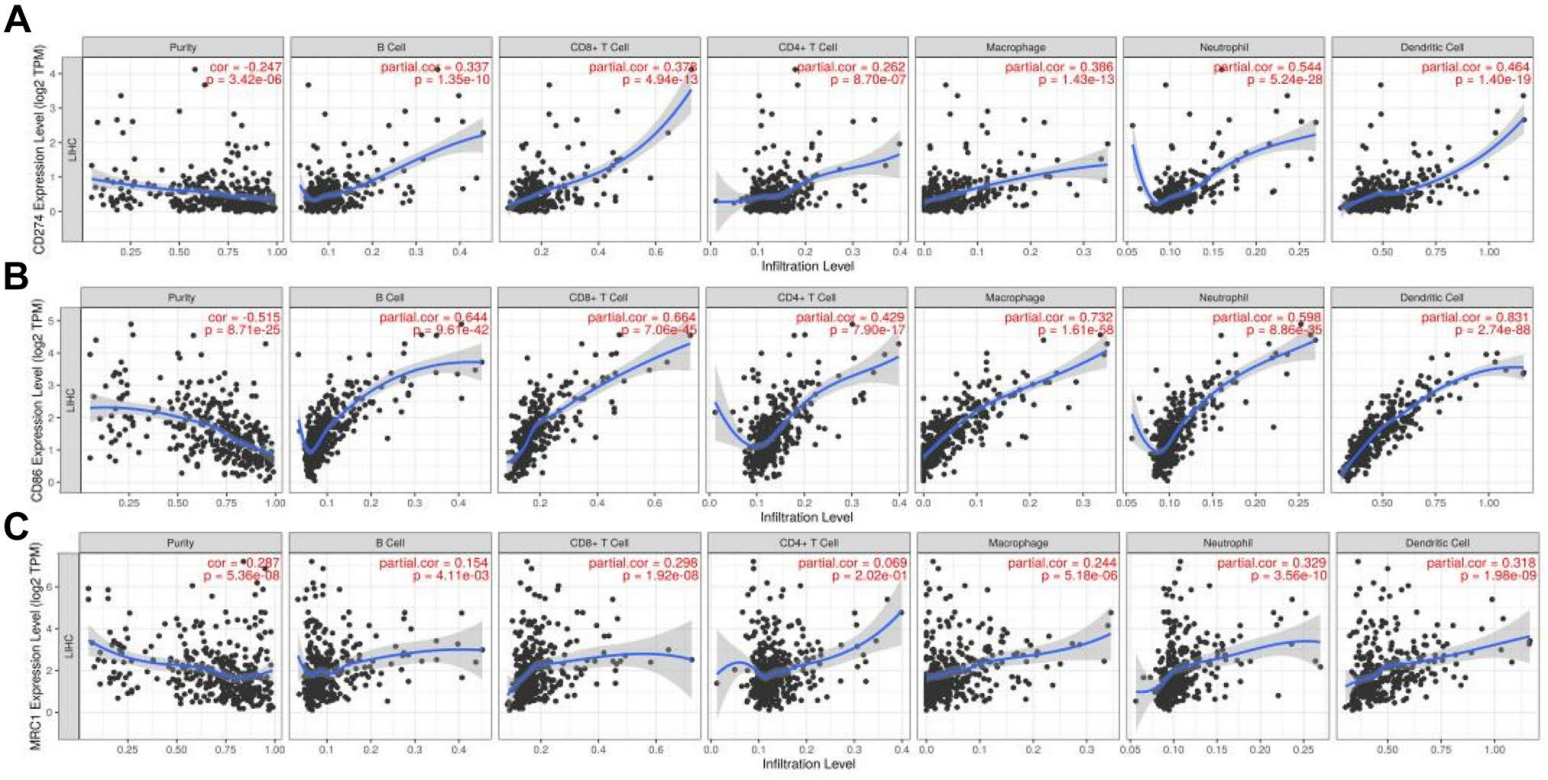
between PD-L1, CD86, and CD206 expression and immune cell infiltration in HCC. **A** Relationship between PD-L1 expression and immune cell infiltration in HCC. **B** Relationship between CD86 expression and immune cell infiltration in HCC. **C** Relationship between CD206 expression and immune cell infiltration in HCC

The relationship between the expression of PD-L1, CD86, and CD206 and the regulation of the purity of immune cell infiltration (B cells, CD4^+^T cells, CD8^+^T cells, DC, neutrophils and macrophages) was hereby investigated using TIMER database. The expression level of PD-L1 was found to be positively correlated with the infiltration level of B cells (*r* = 0.337, *p* = 1.35e − 10), CD8^+^T cells (*r* = 0.378, *p* = 4.94e − 13) and macrophages (*r* = 0.386, *p* = 1.43e − 13). CD4^+^T cells (*r* = 0.429, *p* = 7.90e − 17), while neutrophils (*r* = 0.544, *p* = 5.24e − 28) and dendritic cells (*r* = 0.464, *p* = 1.40e − 19) were significantly increased in HCC, were negatively correlated with tumor purity (*r* =  − 0.247, *P* = 1.40E − 19). *p* = 3.42e − 06), as shown in Fig. 2A. The expression level of CD86 was positively correlated with the infiltration level of B cells (*r* = 0.644, *p* = 9.61e − 42), CD8^+^T cells (*r* = 0.664, *p* = 7.06e − 45), CD4^+^T cells (*r* = 0.429, *p* = 7.90e − 17), and macrophages (*r* = 0.732, *P* = 1.61e—58). Meanwhile, neutrophils (*r* = 0.598, *p* = 8.86e − 35) and dendritic cells (*r* = 0.831, *p* = 2.74e − 88) were significantly increased in HCC, but were negatively correlated with tumor purity (*r* =  − 0.515, *p* = 8.71e − 25), as shown in Fig. 2B. Similarly, the expression level of CD206 was positively correlated with the infiltration level of B cells (*r* = 0.154, *p* = 4.11e − 03), CD8^+^T cells (*r* = 0.298, *p* = 1.92e − 08), CD4^+^T cells (*r* = 0.069, *p* = 2.02e − 01) and macrophages (*r* = 0.244, *P* = 5.18 e—06), while neutrophils (*r* = 0.329, *p* = 3.56e − 10) and dendritic cells (*r* = 0.318, *p* = 1.98e − 09) were significantly increased in HCC, but were negatively correlated with tumor purity (*r* =  − 0.287, *p* = 5.36e − 08), as shown in Fig. 2C.

### Expression of CD86, CD206, and PD-L1 in tumor-associated macrophages in cancer tissues and adjacent tissues of patients with hepatocellular carcinoma

**Fig. 3:**
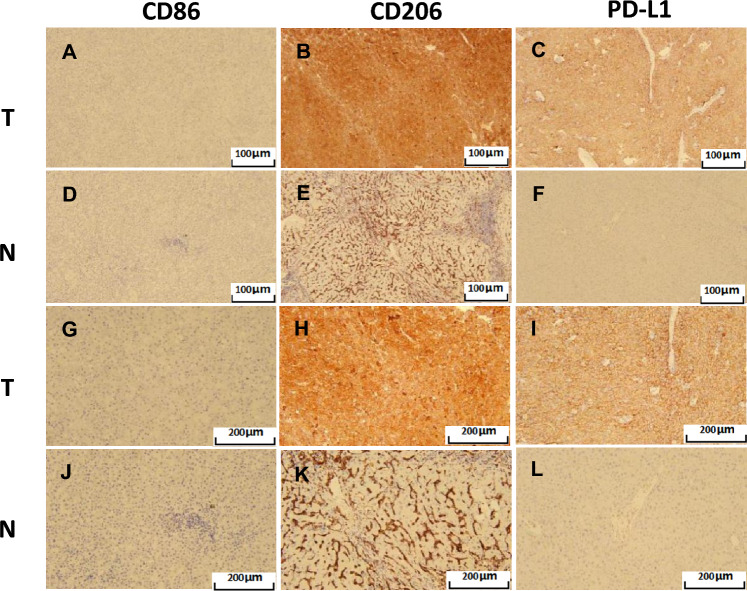
Immunohistochemical staining of CD86, CD206, and PD-L1 in liver cancer tissues and adjacent tissues. **A-C** Immunohistochemical staining of CD86, CD206, and PD-L1 in HCC tissues (SP × 100). **D ~ F** Immunohistochemical staining of CD86, CD206, and PD-L1 in adjacent tissues (SP × 100). **G ~ I** Immunohistochemical staining of CD86, CD206 and PD-L1 in liver cancer tissues (SP × 200). **G ~ I** Immunohistochemical staining of CD86, CD206, and PD-L1 in adjacent tissues (SP × 200)

**Fig. 4:**
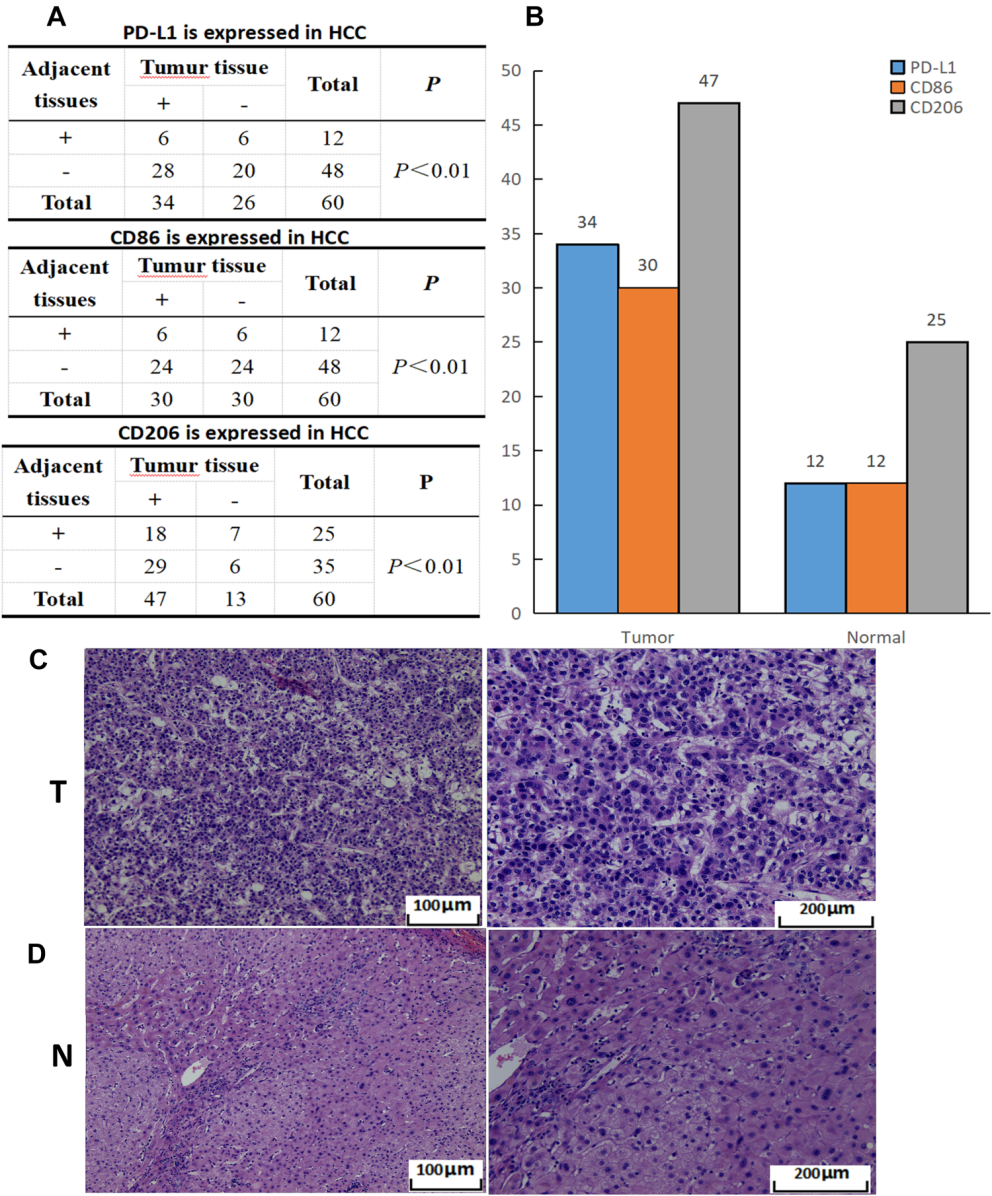
The expression statistics of PD-L1, CD86, and CD206 in liver cancer tissues and adjacent tissues. **A** The differential expressions of PD-L1, CD86, and CD206 in cancer tissues and adjacent tissues.(P < 0.05 were statistically significant). **B** Bar chart of PD-L1, CD86, and CD206 expression levels in cancer tissues and adjacent tissues. **C** H&E staining of liver cancer tissue. **D** H&E staining of liver tissue

Immunohistochemical examination of cancer tissues and adjacent tissues from 60 patients with liver cancer showed that CD86, CD206, and PD-L1 were significantly stained in cancer tissues, as shown in Fig. 3, while statistical analysis showed a positive expression rate of PD-L1 of 57% in cancer tissues and 12% in adjacent tissues. The positive expression rate of CD86 was 50% in cancer tissues and 20% in adjacent tissues, while that of CD206 in cancer tissues was 78.3% and that in adjacent tissues was 41.7%, presenting significant differences, as shown in Fig. 4. H&E staining expression profiles of liver cancer and paracancer tissues are shown in Fig. 4C-D.

### Relationship between the expression of CD86, CD206, and PD-L1 and clinicopathological features in patients with hepatocellular carcinoma

The correlation analysis between the expression levels of CD86, CD206, and PD-L1 in cancer tissues of 60 patients with HCC and the clinicopathological features indicated the correlation of the expression of PD-L1 with the degree of tumor differentiation (*χ2* = 7.855, *P* = 0.02). The expression level of CD206 was correlated with gender (*χ2* = 4.832, *P* = 0.028) and whether patients had hepatitis before surgery (*χ2* = 9.624, *P* = 0.002), as shown in Table 1. Logistic regression analysis of PD-L1 expression and clinicopathological features in tumor tissues of patients with liver cancer demonstrated the degree of tumor differentiation as a risk factor for PD-L1 expression, which was positively correlated with its expression level, as shown in Table 2. Similarly, gender and preoperative hepatitis were found to be risk factors for CD206 expression, which were positively correlated with CD206 expression, as shown in Table 3.

**Table 1:** Correlation between the expression of PD-L1, CD86, and CD206 and clinicopathological features in hepatocellular carcinoma

Characteristics	PD-L1 expression				CD86 expression				CD206 expression			
	Low	High	*χ*^*2*^	*P*	Low	High	*χ*^*2*^	*P*	Low	High	*χ*^*2*^	*P*
Gender												
Male	19	27	0.331	0.656	23	23	0.00	1.00	7	39	4.831	**0.028**
Female	7	7			7	7			6	8		
Age												
< 60	13	21	0.830	0.362	18	16	0.271	0.602	9	25	1.067	0.302
≥ 60	13	13			12	14			4	22		
Tumor size(cm)												
< 5	13	16	0.051	0.821	14	15	0.067	0.796	6	23	0.032	0.859
≥ 5	13	18			16	15			7	24		
Tumor capsule												
Yes	13	16	0.051	0.821	14	15	0.067	0.796	6	23	0.032	0.859
No	13	18			16	15			7	24		
Number of tumors												
Single	22	24	1.621	0.203	22	24	0.373	0.542	9	37	0.513	0.474
Multiple	4	10			8	6			4	10		
Vascular invasion												
Yes	5	12	1.872	0.171	9	8	0.082	0.774	5	12	0.838	0.360
No	21	22			21	22			8	35		
Portal vein tumor thrombus												
Yes	14	16	0.271	0.602	14	16	0.267	0.606	7	23	0.098	0.754
No	12	18			16	14			6	24		
Cirrhosis												
Yes	15	16	0.667	0.414	15	16	0.067	0.796	6	25	0.202	0.653
No	11	18			15	14			7	22		
AFP(ug/L)												
< 13.4	16	11	5.287	0.071	14	13	0.085	0.959	6	21	1.852	0.396
13.4 ≤ AFP < 400	7	14			10	11			6	15		
≥ 400	3	9			6	6			1	11		
Hepatitis												
Yes	19	21	0.848	0.357	21	19	0.300	0.584	4	36	9.624	**0.002**
No	7	13			9	11			9	11		
Differentiation degree												
Low	5	12	7.855	**0.02**	7	10	1.744	0.418	5	12	1.075	0.584
Medium	14	21			20	15			6	29		
High	7	1			3	5			2	6		
AJCC stage												
I	14	10	3.716	0.156	11	13	1.217	0.544	3	21	2.111	0.348
II	5	11			7	7			4	12		
III	7	13			12	8			6	14		

*Statistically significant

The bold values were considered statistically significant

**Table 2:** Logistic regression analysis of the relationship between clinicopathological characteristics and the expression of PD-L1 in hepatocellular carcinoma

Characteristics	*β*	SE	OR	95%CI	*P* value
Differentiation degree (low vs. medium)	2.821	1.194	16.80	1.617 ~ 17.519	0.018
Differentiation degree (medium vs. high)	2.351	1.123	10.5	1.161 ~ 94.925	0.036

**Table 3:** Logistic regression analysis of the relationship between clinicopathological characteristics and the expression of CD206 in hepatocellular carcinoma

Characteristics	*β*	SE	OR	95%CI	*P*
Gender (male vs. female)	1.436	0.757	4.205	0.954 ~ 18.542	0.048
Hepatitis (yes vs. no)	2.001	0.718	7.394	1.785 ~ 30.629	0.006

### Effect of PD-L1, CD86, and CD206 expression on the overall survival and progression-free survival in patients with hepatocellular carcinoma

**Fig. 5:**
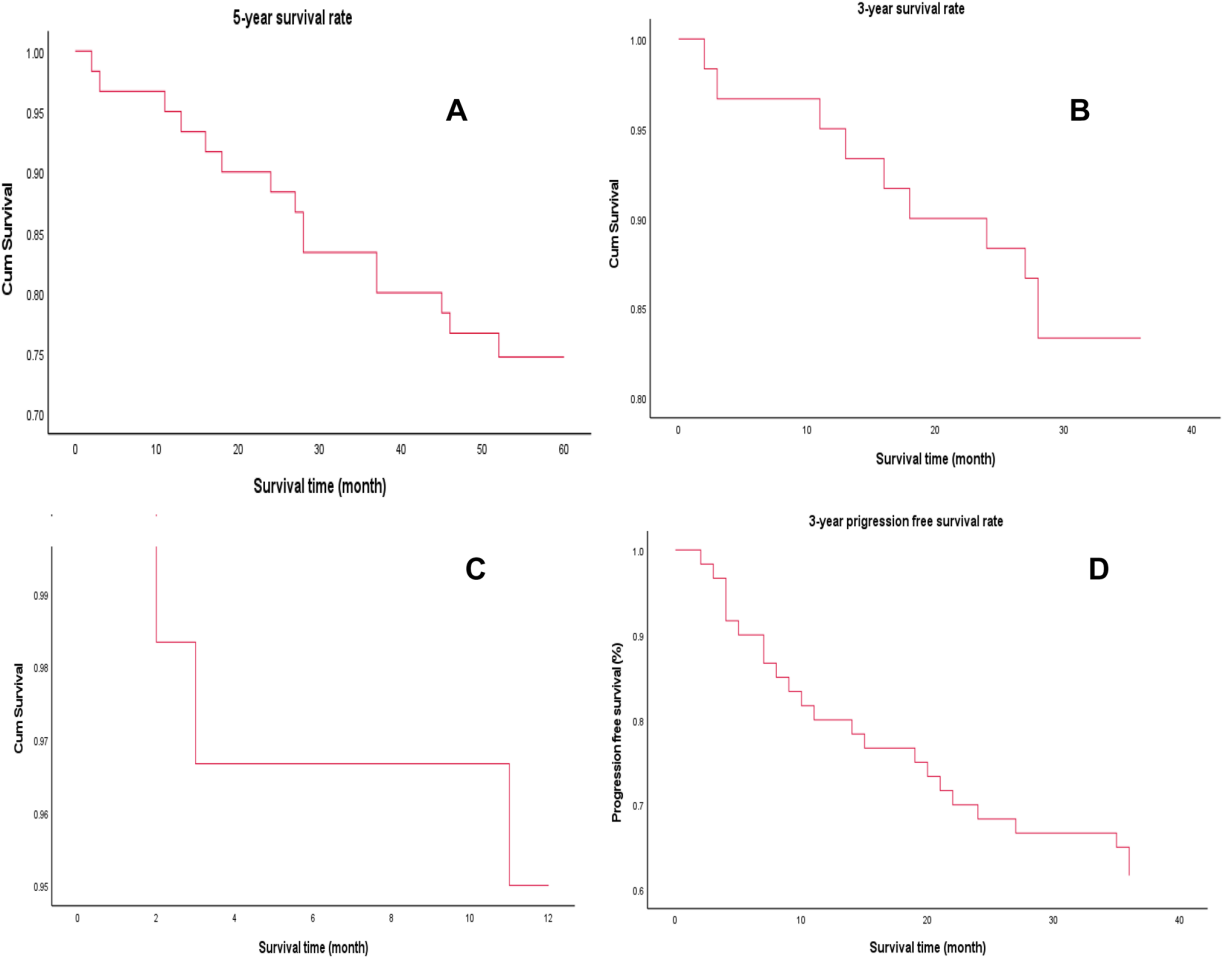
Survival analysis of patients with liver cancer. **A** 5-year overall survival rate of patients with liver cancer. **B** 3-year overall survival rate of patients with liver cancer. **C** 1-year overall survival rate of patients with liver cancer. **D** 3-year progression-free survival rate of patients with liver cancer

**Fig. 6:**
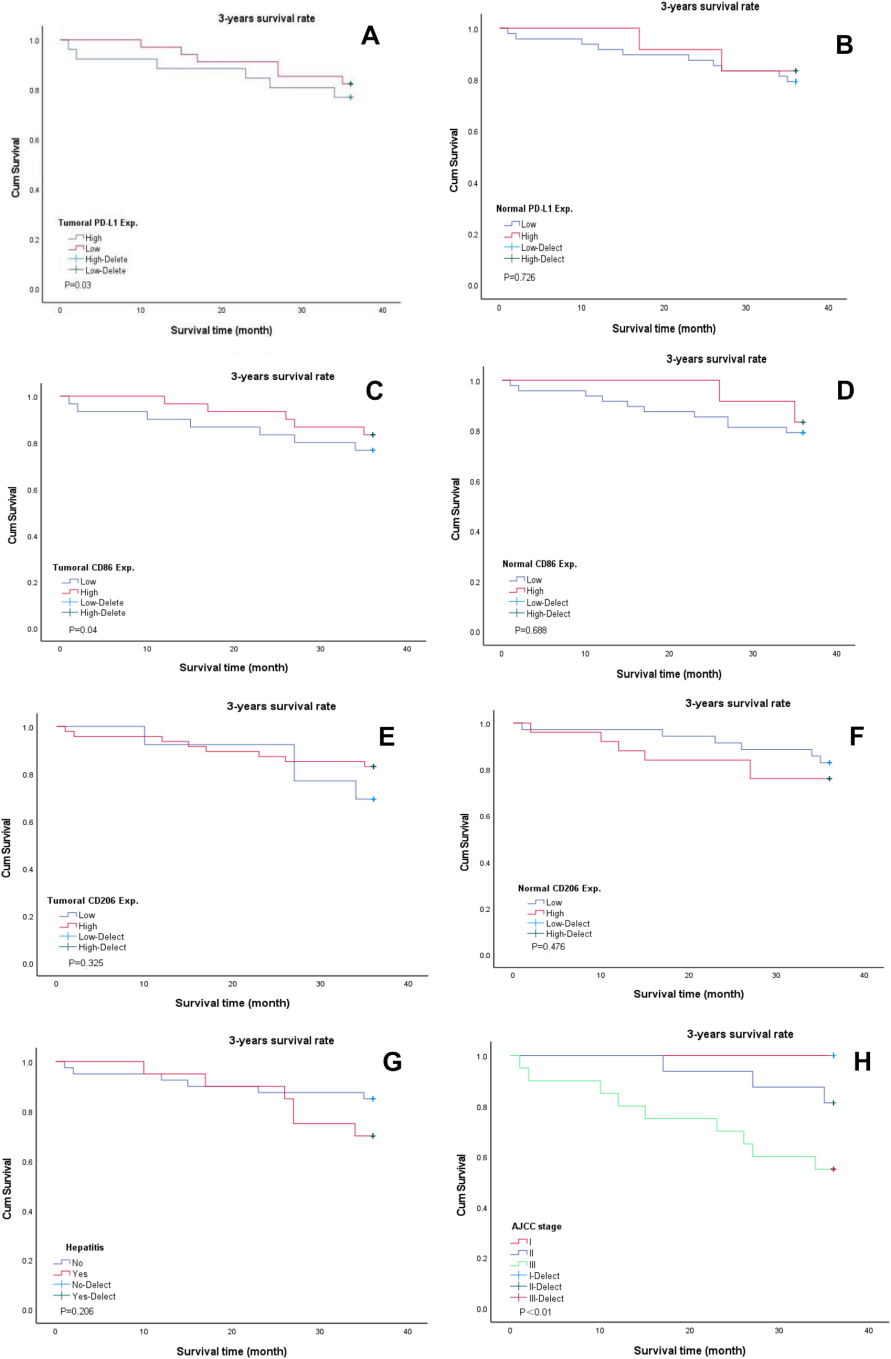
Kaplan–Meier survival analysis of patients with liver cancer. **A** The relationship between PD-L1 expression and 3-year overall survival in cancer tissues. **B** The relationship between the expression of PD-L1 in adjacent tissues and the 3-year overall survival. **C** The relationship between CD86 expression and 3-year overall survival in cancer tissues. **D** The relationship between the expression of CD86 in adjacent tissues and the 3-year overall survival. **E** The relationship between CD206 expression and 3-year overall survival in cancer tissues. **F** The relationship between the expression of CD206 in adjacent tissues and the 3-year overall survival. **G** The relationship between preoperative hepatitis and 3-year overall survival. **H** The relationship between AJCC stage and 3-year overall survival rate

Sixty patients with liver cancer were followed-up after surgery. The shortest survival time was 1 month, while the longest was 70 months. The 1-, 2-, and 3-year overall survival (OS) rates were 93.3%, 83.3%, and 75%, respectively, and the 3-year progression-free survival rate was 61.7%, as shown in Fig. 5. The 3-year overall survival rate of patients with high expression of PD-L1 in liver cancer tissues was 76.9%, while that of those with low expression was 82.4%, presenting significant differences (*P* = 0.03), as shown in Fig. 6A. Besides, no correlation was observed between PD-L1 expression and prognosis in adjacent to cancer tissue, as shown in Fig. 6B. Analysis of CD86 in HCC tissues showed that the 3-year overall survival rate of patients with high expression of CD86 was 83.3%, while that of those with low expression of CD86 was 79.2%, showing significant difference (*P* = 0.04), as shown in Fig. 6C. However, there was no correlation between the expression of CD86 in adjacent tissues and the prognosis, as shown in Fig. 6D. No correlation was observed from the survival analysis on the expression of CD206 in cancer tissues and adjacent tissues and whether they had hepatitis before surgery, as shown in Fig. 6E–G. As shown in Fig. 6H, survival analysis of AJCC stage showed that the 3-year survival rates of patients with stage I, II, and III were 100%, 81.3%, and 55.2%, respectively, showing differences. Cox multivariate analysis showed tumor AJCC stage (OR = 11.841, 95%CI: 2.589–54.16, *P* = 0.001) and preoperative hepatitis (OR = 5.427, 95%CI: 1.084–27.175, *P* = 0.04), and the expression level of PD-L1 in paracancer tissues (OR = 7.172, 95%CI: 1.405–36.606, *P* = 0.018) was an independent risk factor affecting survival of patients after radical hepatoma surgery, as shown in Table 4.

**Table 4:** Cox regression analysis of prognostic factors in patients with hepatocellular carcinoma after radical resection

Characteristics	*β*	SE	OR	95%CI	*P* value
AJCC stage (I *vs.* II ~ III)	2.472	0.776	11.841	2.589 ~ 54.16	0.001
Hepatitis (yes *vs.* no)	1.691	0.822	5.427	1.084 ~ 27.175	0.040
PD-L1 in tissues (high *vs.* low)	1.970	0.832	7.172	1.405 ~ 36.606	0.018

### Construction and validation of the prognostic nomogram of patients with liver cancer

**Fig. 7:**
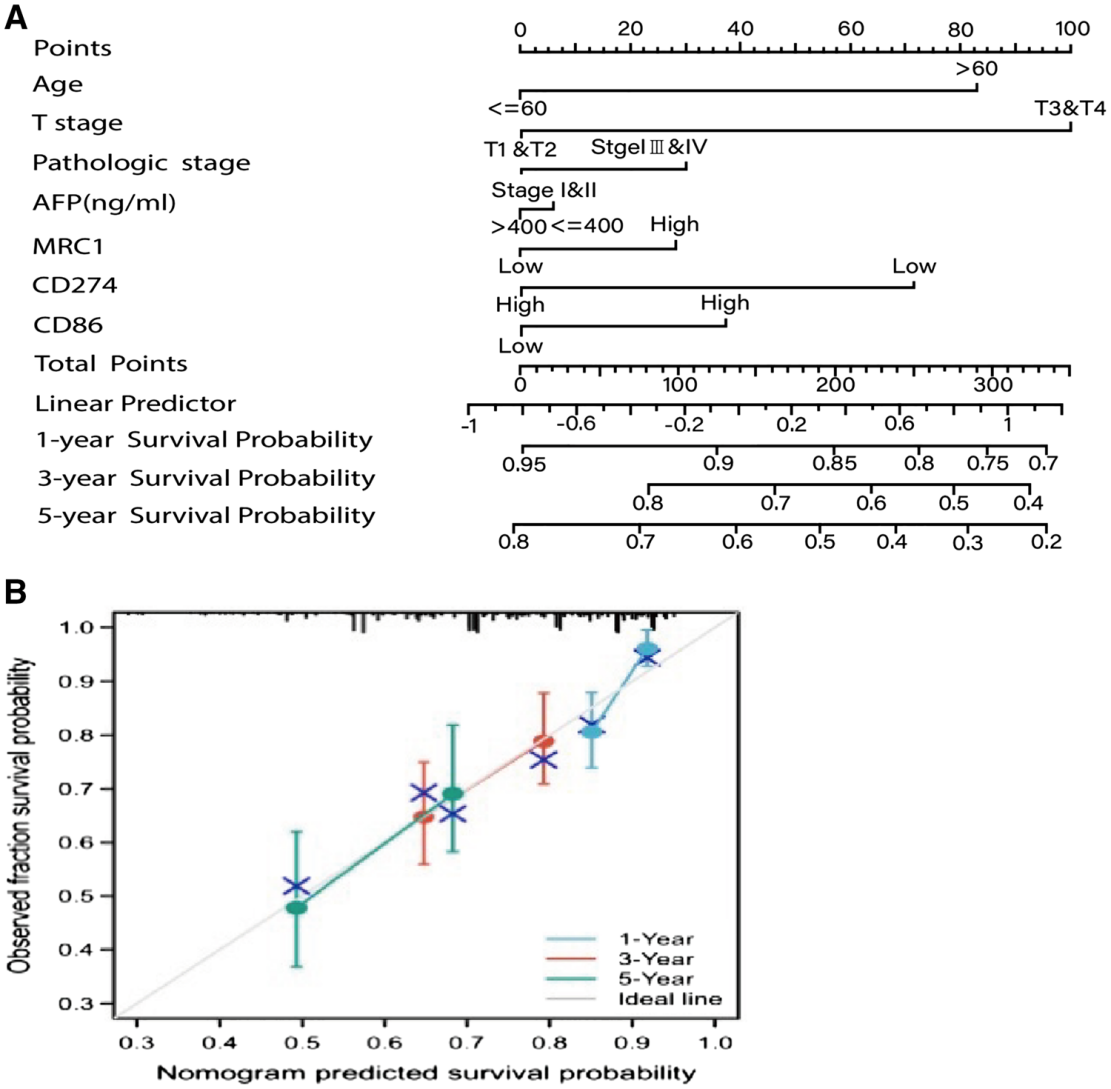
Prognostic prediction model for hepatocellular carcinoma. **A** In the prediction model, the score of each factor is obtained according to the upper scale, and the total score is obtained by adding the score of each factor. From the overall score downward, the corresponding 3- and 5-year overall survival rates were obtained. **B** The comparison of 3- and 5-year overall survival plots predicted by the rotigrams with the observed 3- and 5-year overall survival

According to the results of multivariate analysis, the prognosis prediction model of liver cancer was established using R software. In the nomogram, age ≤ 60 was 0 point, while age > 60 was 80 points. Tumor stages T1 and T2 were 0 point, T3 and T4 were 100 points. The pathological stage of the tumor was 0 for stage I and II, and 30 for stage II and IV. AFP ≤ 400 indicated a score of 0, while AFP > 400 represented a score of 12. The low expression of PD-L1 was 0, and the high expression was 28. The high expression of CD206 was 0, while the low expression was 72. The low expression of CD86 was 0, while the high expression was 38. The higher the total score in the nomogram was, the lower the OS in the corresponding 3 and 5 years would be, as shown in Fig. 7A. The test results showed that the C-index of the line chart model was 0.742, indicating the high accuracy of the model.

The independent samples of this study were used to test the consistency of the column chart, and the 3-year and 5-year OS predicted by the model was found to be in good agreement with the actual 3-year and 5-year OS. The Bootstrap method (repeated sampling for 1 000 times) was used to verify the established line graph. The X-axis of the correction curve represented the survival rate predicted by the line graph, while the Y-axis represented the actual survival rate of patients. Meanwhile, the accuracy of the line graph was represented by the consistency between the solid and dashed lines in the graph. The C-index of internal verification was 0.762 (95%CI: 0.744 ~ 0.7780), and the C digit of external verification was 0.7787 (95%CI: 0.767 ~ 0.7806), indicating the good predictive value of the line graph. The predicted values of the calibration charts of 1-year, 3-year, and 5-year CSS in the two groups were in good agreement with the actual observed values, as shown in Fig. 7B.

### Functional prediction and protein interaction analysis of PD-L1, CD86, and CD206

**Fig. 8:**
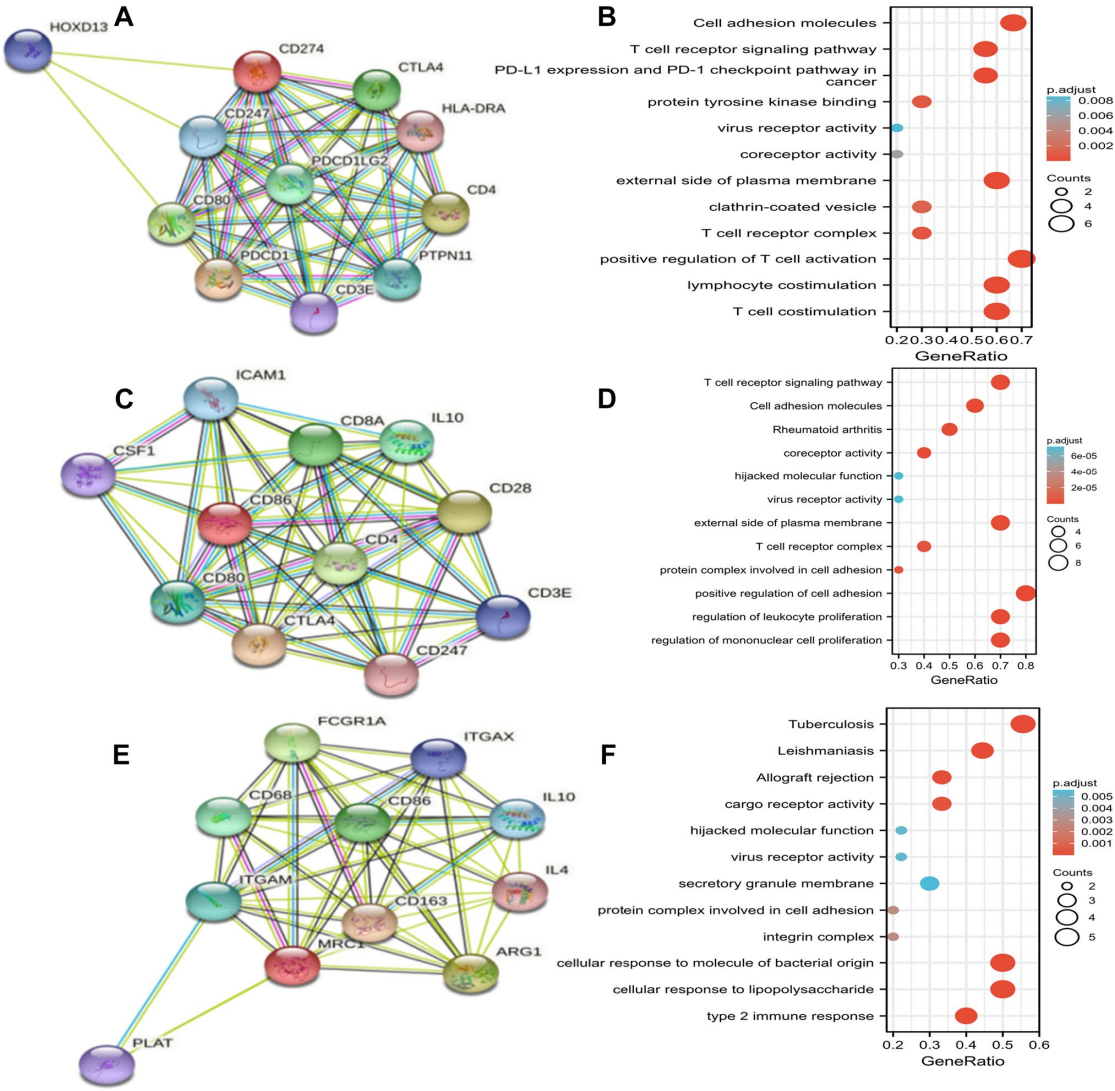
Protein–protein interaction and functional enrichment analysis of PD-L1, CD86, and CD206. **A** Schematic diagram of functional analysis of protein–protein interaction in PD-L1.** B** PD-L Functional enrichment analysis. **C** Schematic diagram of functional analysis of protein–protein interaction in CD86. **D** CD86 functional enrichment analysis. **E** Schematic diagram of functional analysis of protein–protein interaction in CD206. **F** CD206 functional enrichment analysis

Based on STRING database, the PPI networks of PD-L1, CD86, and CD206 were constructed, respectively, and the top ten functionally interacting proteins with high connectivity were selected, among which, PD-L1-related genes were CD247, CD80, CTLA4, PDC1LG2, PDCD1, HLA-DRA, CD3E, CD4, PTPN11, and HOXD13, as shown in Fig. 8A, while CD86-related genes were ICAM1, CD80, CD8A, CTLA4, IL10, CTLA4, CD4, CD28, CD247, CD3E, and CSF1, as shown in Fig. 8C. Besides, CD206-related genes were CD68, FCGR1A, ITGAM, PLAT, ITGAX, CD86, CD163, IL10, IL4, and ARG1, as shown in Fig. 8E. GO enrichment analysis involved three main functions, namely biological process function, cellular component function, and molecular function (*P* < 0.05). KEGG pathway and GO analysis showed that PD-L1 promoted significant enrichment in T cell aggregation, lymphocyte aggregation, and other aspects of biological processes. In terms of cell components, PD-L1 participated in T cell antigen receptor CD3 complex, cell membrane and other functions, as shown in Fig. 8B and Table 5. CD86 was significantly enriched in the positive regulation of cell adhesion, regulation of mononuclear cell proliferation, regulation of leukocyte proliferation, and transduction of T cell receptor signaling pathway, as shown in Fig. 8D and Table 6. Similarly, KEGG pathway analysis of CD206 presented significant enrichment in type 2 immune response, cellular response to LPS, cellular response to LPS, and cellular response to LPS involved in cellular response to LPS, as shown in Fig. 8F.

**Table 5:** GO and KEGG enrichment analyses of PD-L1 and functional partner genes in hepatocellular carcinoma

Ontology	ID	Description	*P* value
BP	GO:0031295	T cell co-stimulation	1.15e-13
BP	GO:0031294	Lymphocyte co-stimulation	1.28e-13
BP	GO:0050870	Positive regulation of T cell activation	1.83e-12
CC	GO:0009897	External side of plasma membrane	1.19e-08
CC	GO:0042101	T cell receptor complex	3.03e-05
CC	GO:0030136	Clathrin-coated vesicle	9.75e-05
MF	GO:1,990,782	Protein tyrosine kinase binding	1.64e-05
MF	GO:0015026	Co-receptor activity	2.68e-04
MF	GO:0001618	Virus receptor activity	7.60e-04
KEGG	hsa04514	Cell adhesion molecules	2.86e-09
KEGG	hsa05235	PD-L1 expression and PD-1 checkpoint pathway in cancer	1.77e-08
KEGG	hsa04660	T cell receptor signaling pathway	3.89e-08

**Table 6:** GO and KEGG enrichment analyses of CD86 and functional partner genes in hepatocellular carcinoma

Ontology	ID	Description	*P* value
BP	GO:0045785	Positive regulation of cell adhesion	1.91e-12
BP	GO:0032944	Regulation of mononuclear cell proliferation	2.32e-12
BP	GO:0070663	Regulation of leukocyte proliferation	3.56e-12
CC	GO:0009897	External side of plasma membrane	1.35e-10
CC	GO:0042101	T cell receptor complex	3.35e-07
CC	GO:0098636	Protein complex involved in cell adhesion	5.58e-07
MF	GO:0015026	Co-receptor activity	6.90e-09
MF	GO:0001618	Virus receptor activity	8.25e-06
MF	GO:0104005	Hijacked molecular function	8.25e-06
KEGG	hsa04660	T cell receptor signaling pathway	5.57e-12
KEGG	hsa04514	Cell adhesion molecules	7.05e-09
KEGG	hsa05323	Rheumatoid arthritis	4.38e-08

## Discussion

Tumor microenvironment (TME) is a dynamic system regulated by cell-to-cell communication and is closely related to tumor development and metastasis (Wu et al. 2021; Lan et al. 2019; Quail and Joyce 2013; Bakir et al. 2020). Macrophages, as the main stromal cells in TME, are highly plastic and have different phenotypes under different stimuli, including M1 (tumor suppressor) and M2 (tumor promoter) (Witherel et al. 2021). TAMs are generally considered M2-type macrophages with high expression of CD206, Arg-1, IL10, and TGF-β (Dawei and Rong 2019; Qian and Pollard 2010). According to previous studies, M1 macrophages have been considered to play an inhibitory role in tumor growth, while M2 macrophages promote tumor growth (Hinshaw and Shevde 2019; Sharifi et al. 2019). Jiang et al. even reported that M1 macrophages inhibited the migration and invasion of esophageal squamous cell carcinoma cells (Jiang et al. 2021). Additionally, M1 macrophages inhibit the proliferation, induce apoptosis of tumor cells in lung cancer, and play an anti-tumor role (Li et al. 2020). Herein, it was also found that CD86 (M1 macrophages) significantly infiltrated in cancer tissues. Prognostic analysis of the expression of CD86 in cancer tissues showed that CD86 could inhibit tumor progression and prolong the overall survival time of patients for 3 years (*P* = 0.04), which further verified the effect of M1 macrophages in inhibiting tumor growth. However, the tumor-promoting effects of M1 macrophages have been validated in recent years. Jin et al. observed that M1 macrophages could promote the invasion of brain glioma U251 cells (Jin et al. 2019). Zhuo Chen et al. detected that M1-type macrophages could induce EMT in breast cancer cells T47-D and MCF-7, and enhance the migration and invasion ability of the cells. Targeting M1 macrophages might inhibit EMT and limit the invasion potential of breast cancer (Chen et al. 2022). However, the effect of M1 macrophages on tumor promotion or inhibition is still controversial at present, and the role of M1 macrophages in the occurrence and progression of bladder cancer is still unclear, indicating the necessity of more basic studies.

Programmed death-1 (PD-1) is a type I transmembrane protein mainly expressed in mature T cells, B cells, macrophages, and NK cells. Its ligand PD-L1 is inductively expressed in T cells, B cells, monocytes, and many types of tumor cells, such as lung cancer, liver cancer, and malignant melanoma (Yuan et al. 2021). Under normal physiological conditions, PD-L1 expressed on the surface of tissue cells, endothelial cells, and immune cells binds to PD-1 on the surface of activated T cells, which inhibits excessive activation of T cells and induces apoptosis of T cells, thus maintaining a certain dynamic balance of the immune response of the body (Dougan 2017). After carcinogenesis, tumor cells promote the upregulation of PD-L1 expression through a variety of mechanisms. A large number of PD-1 molecules on the surface of T cells inhibit the proliferation and activation of CD4^+^T cells and CD8^+^T cells, and some activated cytokines (such as IFN-γ) are produced to break the homeostasis of immune response, which facilitates tumor cells to evade the surveillance of the body's immune system and promotes tumor progression (Butte et al. 2008; Zhu et al. 2018). Herein, bioinformatics analysis found that the expression levels of PD-L1, CD86, and CD206 were downregulated in tumor tissues, while immunohistochemical staining data showed that PD-L1, CD86, and CD206 were overexpressed in tumor tissues. After analysis, PD-L1, CD86, and CD206 were considered to be overexpressed in tumor tissues. The expressions of PD-L1, CD86, and CD206 in hepatocellular carcinoma cells were lower than those in the adjacent microenvironment. However, the hereby taken cancer tissue was normal liver tissue > 1 cm away from the tumor, so the expression level in the cancer tissue was higher than that in the control group (normal liver tissue). The difference between the results of bioinformatics and those of immunohistochemistry in this paper was that there might be differences in the selection of the included standard control group, which resulted in different results. The present study also found that PD-L1 was significantly overexpressed in cancer tissues, which promoted tumor progression and affected the prognosis of patients, also consistent with the results reported in the literature.

Liver is a special immune-tolerant organ that can effectively evade immune response. However, immunotherapy can enhance the immune response of the body, break immune tolerance, and then recognize and kill tumor cells (Sung et al. 2021). In recent years, tumor immunotherapy represented by immune checkpoint inhibitors (ICIs) has made breakthrough progress, prolongating the survival of patients. Meanwhile, some patients can even be transformed from unresectable to radical resectable, bringing new light to the treatment of tumors (Dimitri et al. 2020). In this study, the bioinformation analysis first found that PD-L1, CD86, and CD206 were differentially expressed in HCC and correlated with the immune infiltration of tumor cells. To further confirm the finding, immunohistochemical detection of PD-L1, CD86, and CD206 in cancer tissues and adjacent tissues of 60 HCC patients was conducted by the research group, and it was found that PD-L1, CD206, and CD86 were significantly overexpressed in cancer tissues, which might be attributed to the suppression of immune response caused by PD-L1 overexpression in the immune microenvironment of HCC (Zhang et al. 2018). Generally, the application of PD-1 antibody can improve the immune suppression state of the body and enhance the anti-tumor immune effect of the body, which has achieved certain efficacy in some solid tumors. However, in the immunotherapy of liver cancer, the anti-PD-1 efficacy is as low as only 15–25%, which may be related to the special immune microenvironment of liver cancer, and the specific mechanism is still unclear (Pinero et al. 2020). To improve the immune response rate of patients with liver cancer and increase the efficacy of anti-PD-1, it is urgent to further study the immune microenvironment of liver cancer and find more effective tumor therapeutic targets, so as to achieve the purpose of precise treatment.

To further explore the biological functions of PD-L1, CD86, and CD206, GO and KEGG enrichment analysis were hereby carried out. PD-L1 is mainly involved in T cell activation, aggregation, and lymphocyte activation in tumor microenvironment, while CD86 is mainly involved in the positive regulation of cell adhesion and the proliferation of leukocytes and monocytes, and CD206 is mainly involved in type 2 immune response and protein complex involved in cell adhesion. However, the current study was still subjected to certain limitations. First of all, only the function of each gene was predicted, and the research depth was not sufficient enough. The function and mechanism of tumor immune infiltration should be further explored. Second, in this study, only HCC patients who underwent radical surgery in a single institution were retrospectively analyzed. The value of PD-L1, CD86, and CD206 expression in tumor tissues should be prospectively verified in multicenter studies.

## Conclusion

Herein, the expression of PD-L1, CD86, and CD206 in hepatocellular carcinoma tissues was investigated, and the application value of PD-L1, CD86, and CD206 in prognosis assessment of hepatocellular carcinoma was revealed. PD-L1, CD86, and CD206 might be involved not only in the occurrence and development of HCC, but also in the immune escape of HCC. Therefore, the in-depth study of PD-L1, CD86, and CD206 could not only find biomarkers for prognosis assessment of patients with liver cancer, but also provide new therapeutic targets for the immunotherapy of the patients. However, the current study still had certain limitations, and further mechanistic studies should be conducted to verify the present findings and promote clinical application.

## Data Availability

Data supporting the conclusions of this article are included within the article. The raw datasets used and analysis for the present study are available from the corresponding author upon reasonable request.
